# Postoperative stroke in CABG: a ten-year study of stroke incidence and contributing risk factors in Jordan

**DOI:** 10.25122/jml-2024-0326

**Published:** 2025-07

**Authors:** Emad Hijazi, Nabil Al-Zoubi, Qusai Aljarrah

**Affiliations:** 1 Department of General Surgery and Urology, Faculty of Medicine, Jordan University of Science and Technology, Irbid, Jordan; 2 King Abdullah University Hospital, Irbid, Jordan

**Keywords:** coronary artery bypass grafting, CABG, stroke, surgical skills, complications, CABG, Coronary Artery Bypass Grafting, CEA, Carotid Endarterectomy, CICU, Cardiac Intensive Care Unit, COPD, Chronic Obstructive Pulmonary Disease, CPB, Cardiopulmonary Bypass, CT, Computed Tomography, CVA, Cerebrovascular Accident, IV, Intravenous, LIMA, Left Internal Mammary Artery, LVEF, Left Ventricular Ejection Fraction, OPCAB, Off-Pump Coronary Artery Bypass Grafting, RCT, Randomized Controlled Trial, TCD, Transcranial Doppler Ultrasonography, TIA, Transient Ischemic Attack

## Abstract

Stroke is a serious complication that occurs in approximately 3% of patients undergoing elective coronary artery bypass grafting (CABG). Neurologic dysfunction is a well-researched challenge in cardiac surgery, particularly when cardiopulmonary bypass is used. This study aimed to determine the stroke rate following CABG in a Jordanian population, based on our experience at a low-volume cardiac center. We conducted an observational descriptive study involving 446 patients who underwent CABG between January 2011 and December 2021. Stroke occurred in five patients (1.12%). Among 208 female patients, four developed stroke (1.9%), three of whom had diabetes mellitus (75%), and two had a prior history of transient ischemic attacks (TIAs). In contrast, only one out of 238 male patients experienced a stroke (0.4%); this patient had diabetes but had no history of TIA. The incidence of stroke in female patients was four times higher than in men. These findings suggest that improving surgical techniques, particularly in low-resource or developing settings, may contribute to reducing the risk of postoperative stroke. Strategies such as minimizing aortic manipulation during CABG while utilizing cardiopulmonary bypass could be beneficial, especially in patients with known risk factors for cerebrovascular events.

## INTRODUCTION

Stroke is a serious complication that occurs in approximately 3% of patients undergoing coronary artery bypass grafting (CABG) [1]. Neurologic dysfunction is a well-researched obstacle in cardiac surgery, particularly when cardiopulmonary bypass is used [2]. Microembolus formation is commonly caused by cardiopulmonary bypass (CPB), along with increased permeability of the blood-brain barrier, which has been shown to impair cognitive function [3,4]. On-pump CABG, utilizing a single cross-clamp rather than the double clamp technique, reduces the risk of postoperative stroke [5]. This research was carried out to identify the incidence of stroke after coronary bypass surgery in the Jordanian population.

## MATERIAL AND METHODS

### Study design and setting

A descriptive, observational study was conducted at the Princess Muna Al-Hussein Cardiac Center, located at King Abdullah University Hospital, which is affiliated with the Jordan University of Science and Technology in Irbid, Jordan. Patients who underwent coronary artery bypass grafting between January 2011 and December 2021 were included.

### Inclusion and exclusion criteria

Patients undergoing elective CABG were enrolled. Exclusion criteria included patients with redo CABG, concurrent valve surgery, left ventricular ejection fraction (LVEF) <0.40, and those with carotid artery disease not eligible for stenting or partial revascularization due to stroke risk.

### Neurological evaluation

Neurology consultations were performed, and both CT and MRI were obtained. While CT scans were consistently negative within the first 24 hours, an MRI was used to confirm findings. We typically avoided sending patients for MRI within 24 to 48 hours post-open-heart surgery due to their instability during this period. In all cases, the cause of the neurological deficit was identified as embolic, with no evidence of intracranial bleeding. Therefore, we ruled out hemorrhagic stroke, and no cases of transient ischemic attack (TIA) were observed. The deficits were confirmed as embolic strokes. Routine initial CT scans were only performed for patients who developed neurological deficits. Following the neurologist’s protocol, all patients with suspected deficits underwent a CT within 24–48 hours to rule out bleeding, followed by an MRI to confirm the diagnosis. In cases of transient ischemic attack, the same protocol was applied.

Based on the cardiac surgery follow-up protocol in our institution, all patients were examined preoperatively, post-extubation in the cardiac intensive care unit (CICU), daily during hospitalization, and at follow-up visits 3–4 weeks post-discharge. A fresh external and central neurological deficit was diagnosed as a stroke. The cardiac surgeon identified such deficits immediately after extubation, typically within 5–6 days of hospitalization. Based on neurologist recommendations, a routine CT scan of the head was performed in each suspected case. Carotid artery stenosis was categorized into three levels: no disease (luminal narrowing <50%), moderate disease (>50% but <80% narrowing), and severe disease (80–99% narrowing). These categories were considered clinically insignificant in the context of embolic stroke unless associated with broad or diffuse obstruction [6].

### Surgical technique

All procedures were performed by a single surgeon using conventional CABG techniques. The left internal mammary artery (LIMA) was used as the primary graft. Electrocautery was employed to excise parasternal and pericardial tissues, and a midline sternotomy was performed to access the thoracic cavity. Gentle palpation of the ascending aorta, aided by the application of bone wax, was carried out to identify any areas of calcification and thus avoid those sites during cannulation. The heart was manipulated carefully to prevent trauma, and the aortic cross-clamp was placed with caution, even in the presence of partial but unavoidable calcification. The same cross-clamp was used throughout the procedure and was not repositioned during proximal graft anastomosis, which was performed with a single aortic clamp technique.

Cardiopulmonary bypass (CPB) was established using a two-stage single atrial cannula and a perfusion cannula inserted into the ascending aorta. Standard CPB management protocols were followed, including the use of arterial line filters, membrane oxygenators, and non-pulsatile flow at 2.4 L/min/m^2^. Systemic hypothermia was maintained at 32°C, and the mean arterial pressure was kept above 50 mmHg. Myocardial protection was achieved via intermittent antegrade cardioplegia using high-potassium crystalloid solutions.

Anticoagulation was initiated with 300 U/kg of heparin to maintain an activated clotting time above 480 seconds, and it was reversed postoperatively with protamine sulfate. All patients had left pleural and mediastinal chest drains inserted according to standard protocols. The sternum was closed with stainless steel wires, and the subcutaneous layers were approximated using two layers of dyed Vicryl 2-0 sutures to minimize dead space. Subcuticular skin closure was completed with undyed absorbable Vicryl (polyglactin 910). Prophylactic antibiotic therapy included intravenous TAZOCIN (piperacillin-tazobactam) 4.5 g administered 30 minutes preoperatively, and then every 6 hours postoperatively (adjusted to 2.25 g for patients with renal impairment). For patients with penicillin allergy, vancomycin 1 g IV was administered twice daily, except in cases where chest drains had been removed. Postoperatively, patients were assessed for hemodynamic stability before extubation and were continuously monitored in the CICU. Drains were removed once the output fell below 100 mL over 12 hours.

### Data analysis

Descriptive statistics were performed using the Statistical Package for the Social Sciences (SPSS), version 23.0. Categorical variables were summarized using frequencies and percentages, while continuous variables were described using means and standard deviations or medians and ranges, depending on the normality of their distribution as assessed by the Shapiro-Wilk test. The Chi-squared test or Fisher’s exact test was used for comparisons of categorical variables, as appropriate. Continuous variables were analyzed using independent *t*-tests for normally distributed data and the Wilcoxon rank-sum test when assumptions of normality were violated.

## RESULTS

### CT-carotid angiogram findings and patient characteristics

CT-carotid angiography was performed in 198 patients (44.39%) aged ≥60 years in cases where a clinically significant carotid bruit was documented or when patients had a history suggestive of transient ischemic attacks. It is important to note that intraoperative epiaortic ultrasound was not available at our institution, limiting real-time assessment of ascending aortic atherosclerosis.

A total of 446 consecutive patients undergoing CABG were included in this study. The median age of the cohort was 59.5 years (range, 35–75 years). Of these, 208 patients (47%) were women and 238 (53%) were men. The demographic and clinical characteristics of the study population are summarized in Table 1. Carotid artery stenosis was observed in 163 patients (36.5%). Stroke occurred in five patients (1.12%), all of whom were ≥65 years of age and diagnosed within the first day post-extubation in the CICU. Among female patients, four cases of stroke were recorded (1.9%). Of these, three patients (75%) had diabetes, and two (50%) had a history of TIA with moderate left carotid stenosis as confirmed by CT-carotid angiography. In male patients, only one stroke was recorded (0.4%); this patient also had diabetes but no history of TIA and only non-significant carotid stenosis. No perioperative mortality was reported in either group (Table 1).

**Table 1:** Characteristics of patients (*n* = 446)

Characteristics	*n*	%
Angina pectoris	169	37.9
Left main coronary lesion	102	22.9
History of MI	84	18.8
History of TIA	14	3.1
Carotid artery stenosis	163	36.5
Moderate right stenosis	21	4.7
Moderate left stenosis	34	7.6
Non-significant carotid stenosis	98	22.0
Diabetes mellitus	191	42.8
Male	89/191	47
Female	102/191	53
Chronic renal failure	4	0.9
Peripheral vascular disease	16	3.6
COPD	89	20.0
History of cerebral embolism	11	2.5
Smoking	279	62.6
Steroid use	7	1.6
Reopening for bleeding	4	0.9
Emergency surgery	9	2.0
Dyspnea	199	44.6
Hypertension	202	45.3
Continuous variables	Value	Unit
Age, Median (Range)	59.5	35-75
Body mass index, Mean (SD)	29.5	3.1
LVEF, Mean (SD)	0.43	0.07

We compared the clinical characteristics between men and women (Table 2). Left main coronary artery lesions were significantly more prevalent in male patients (26.89% vs. 18.27%; *P* < 0.001), as were previous myocardial infarction (22.27% vs. 14.9%; *P* = 0.001), chronic obstructive pulmonary disease (COPD) (25.21% vs. 13.94%; *P* < 0.001), and a history of smoking (78.99% vs. 43.75%; *P* < 0.001).

**Table 2:** Comparison of clinical characteristics based on gender

Variables	Females (*n* = 208, %)	Males (*n* = 238, %)	*P* value^*^
Angina pectoris	75 (36.06)	94 (39.5)	0.050
Left main coronary lesion	38 (18.27)	64 (26.89)	<0.001
History of MI	31 (14.9)	53 (22.27)	<0.001
History of TIA	7 (3.37)	7 (2.94)	1.000
Carotid artery stenosis	73 (35.1)	90 (37.82)	0.076
Moderate right stenosis	10 (4.81)	11 (4.62)	1.000
Moderate left stenosis	13 (6.25)	21 (8.82)	0.090
Non-significant carotid stenosis	46 (22.12)	52 (21.85)	0.475
Diabetes mellitus	102 (49.04)	89 (37.39)	0.219
Chronic renal failure	2 (0.96)	2 (0.84)	1.000
Peripheral vascular disease	8 (3.85)	8 (3.36)	1.000
COPD	29 (13.94)	60 (25.21)	<0.001
History of cerebral embolism	6 (2.88)	5 (2.1)	1.000
Smoking	91 (43.75)	188 (78.99)	0.001
Steroid use	3 (1.44)	4 (1.68)	1.000
Reopening for bleeding	1 (0.48)	3 (1.26)	0.480
Emergency surgery	4 (1.92)	5 (2.1)	1.000
Dyspnea	94 (45.19)	105 (44.12)	0.316
Hypertension	99 (47.6)	103 (43.28)	0.765

* Chi-square, Fisher’s exact test

## DISCUSSION

Stroke following coronary artery bypass grafting is typically embolic and is associated with cardiopulmonary bypass, aortic manipulation during cannulation, and proximal anastomosis of bypass grafts [5]. The presence of a heavily calcified aorta, known as a 'porcelain aorta', has a significant impact on surgical decision-making and patient outcomes [6]. According to Amorim *et al*., porcelain aorta is classified based on anatomical location and surgical implications: Type I involves localized circumferential calcification of the ascending aorta—with Type IA being unclampable and Type IB being clampable—while Type II involves calcification of the descending aorta, irrespective of aortic arch involvement [7].

Misfeld *et al*. conducted a meta-analysis of 11,398 patients from eight studies [8]. This systematic review was conducted on all published evidence to compare neurologic complications after aortic manipulation versus off-pump CABG without aortic manipulation. They concluded that among the patients with higher chances of transient ischemic attack or risk for stroke, avoiding aortic manipulation during off-pump CABG reduces neurologic complications as compared to the typical technique, which includes deploying the ascending aorta.

The incidence of stroke in our study was 1.12%, observed in five patients, all aged 65 years or older. In comparison, Kilger *et al*. reported that 7.4% of patients developed stroke post-operation, which is higher than the incidence in the current study [9]. The mean age of patients was similar between the studies. The reason for this age group may be that older individuals are more likely to undergo CABG surgery to address coronary heart disease (CHD) [10]. A 2015 systematic review also reported a stroke incidence of up to 5.7% after CABG, supporting our findings while highlighting variability among populations [11].

Bowles *et al*. used transcranial Doppler ultrasonography (TCD) to compare on-pump and off-pump CABG, reporting a significant reduction in cerebral microemboli with the off-pump technique. However, the clinical relevance of this finding remains uncertain [12]. Similarly, Masabni *et al*. evaluated 1,236 patients using preoperative carotid ultrasound and found that, while the test influenced management in select cases, it did not significantly reduce perioperative stroke risk. As a result, they recommended a more selective approach to preoperative carotid screening [13].

In a recent review, Sirin reported that modifying surgical techniques, such as using alternative cannulation sites (e.g., femoral, innominate, or axillary/subclavian arteries), in-situ pedicle arterial grafts, hypothermic ventricular fibrillation, or relocating proximal anastomoses, can reduce morbidity and mortality in patients with a porcelain aorta undergoing CABG. These strategies are particularly beneficial when severe atherosclerotic disease makes the ascending aorta unsuitable for standard surgical manipulation [14]. Adams *et al*. conducted a retrospective assessment and found no advantage of universal carotid artery duplex screening prior to major heart surgery. They examined 1,499 patients who underwent cardiac surgical procedures from 1999 to 2010 [15]. Patient information included preoperative carotid artery duplex scan results, comorbidities, demographics, previous history of stroke, postoperative stroke site, and details of carotid endarterectomy (CEA) procedures performed before, during, or after cardiac surgery. They concluded that preoperative carotid artery duplex scan screening did not predict extracranial carotid artery disease and that extracranial carotid artery disease did not cause the majority of postoperative strokes. These results suggest that universal carotid artery duplex scan screening is of limited value in analyzing the concerned diseases, while emphasizing the need for a more targeted strategy.

Lin *et al*. found a low relationship between postoperative stroke and preoperative duplex-documented high-grade carotid stenosis [16]. Their findings suggest that the decision to perform preoperative carotid duplex ultrasound should consider the type of open-heart surgery and the presence of preexisting cardiovascular symptoms. Similarly, Sedrakyan *et al*. conducted a meta-analysis and reported that off-pump CABG was associated with a lower risk of stroke compared to conventional CABG using CPB. However, they cautioned that the evidence must be carefully interpreted due to limitations inherent in randomized controlled trials, including the potential for higher reintervention rates, fewer grafts being performed, and technical constraints related to patient selection and surgical expertise [17].

In a study conducted by Lev-Ran *et al.*, 700 consecutive patients undergoing multivessel off-pump CABG between 2000 and 2003 were evaluated to compare outcomes between those who underwent partial aortic clamping and those managed with aortic no-touch techniques [18]. Manual palpation and selective use of epiaortic ultrasonography were employed to assess aortic pathology. The study found that avoiding partial aortic clamping resulted in superior neurologic outcomes, supporting the benefits of no-touch aortic techniques in reducing embolic risk. Despite the positive findings, technical challenges may limit the widespread implementation of this approach. In this study, to measure atheromatous disease, routine epiaortic ultrasound of the ascending aorta was not performed, and instead, a selective CT-carotid angiogram was conducted. Similarly, Kapetanakis *et al*. examined 7,272 patients who underwent isolated CABG between 1998 and 2002, stratified by the degree of aortic manipulation: no aortic manipulation (OPCAB; *n* = 476), tangential clamping only (OPCAB; *n* = 2,527), and full plus tangential clamping (on-pump CABG; *n* = 4,269) [19]. Their findings displayed a relationship between aortic manipulation and postoperative stroke, with increasing levels of aortic manipulation associated with a higher incidence of cerebrovascular accidents (CVA). Patients who underwent both full and tangential clamping were 1.8 times more likely to suffer a stroke compared to those with no manipulation, while tangential clamping alone was associated with a 1.7-fold increased risk. These findings underscore the association between aortic manipulation and postoperative cerebrovascular complications, emphasizing the value of minimally manipulative strategies when feasible.

Our study observed a higher incidence of stroke in female patients compared to males, a finding consistent with several previous studies [20-26]. The risk of perioperative stroke increases with progressive age, prior neurological events, diabetes mellitus, and carotid artery stenosis. The risk of thromboembolism or atheroembolism and/or impaired cerebral blood flow can be controlled by recognizing patients with extensive cerebrovascular disease [27-30]. Furthermore, the anaortic off-pump coronary artery bypass approach (OPCAB), which employs composite and in situ grafts while avoiding manipulation of the ascending aorta, has been proposed as a strategy to reduce stroke risk. However, successful implementation of anaortic techniques requires significant expertise and well-coordinated surgical teams to ensure optimal outcomes [31].

When comparing male and female patients, we also found notable gender differences. Left main coronary artery disease was more prevalent among men, as was a history of myocardial infarction (MI), suggesting a higher burden of prior cardiac events. Smoking and COPD were also significantly more common in males. While it is well-established that smoking significantly increases the risk of COPD and smoking rates tend to be higher in men, a study by Chung *et al*. showed that female smokers are at higher risk of developing COPD compared to male smokers [32-34].

Our study has several limitations. First, it was conducted at a single center, which may limit the generalizability of our findings to other populations. Second, we did not include intraoperative epiaortic ultrasound to assess atherosclerosis in the ascending aorta, which may result in undetected cases of aortic calcification, which is a potential contributor to stroke. Additionally, we did not include long-term follow-up, which limits the ability to assess delayed stroke or cognitive dysfunction postoperatively. Lastly, the relatively small number of stroke cases (*n* = 5) limits the statistical power to identify significant risk factors or associations.

## CONCLUSION

In conclusion, our findings align with existing literature regarding the incidence and risk factors associated with stroke following coronary artery bypass grafting. In our cohort, stroke occurred in 1.1% of patients, with a higher incidence observed in women. Avoidance of vigorous aortic manipulation during CABG using cardiopulmonary bypass, with single cross-clamp proximal anastomosis, may decrease the risk of post-operative stroke, in the absence of epiaortic ultrasound, especially in patients with higher stroke risk. Surgical precision and careful dissection techniques are critical in minimizing complications. Future studies with a larger sample size and the use of inferential statistics are needed in this context.

## Data Availability

The datasets used and analyzed during the current study are available from the corresponding author upon reasonable request.
